# Therapeutic efficacy of tylvalosin combined with *Poria cocos* polysaccharides against porcine reproductive and respiratory syndrome

**DOI:** 10.3389/fvets.2023.1242146

**Published:** 2023-08-07

**Authors:** Hong Shi, Wentao Luo, Shuaiyang Wang, Jun Dai, Cuilan Chen, Shuo Li, Jie Liu, Weiyuan Zhang, Qi Huang, Rui Zhou

**Affiliations:** ^1^National Key Laboratory of Agricultural Microbiology, College of Veterinary Medicine, Huazhong Agricultural University, Wuhan, China; ^2^Hubei Provincial Bioengineering Technology Research Center for Animal Health Products, Yingcheng, China; ^3^International Research Center for Animal Disease, Ministry of Science and Technology of China, Wuhan, China; ^4^Cooperative Innovation Center of Sustainable Pig Production, Wuhan, China; ^5^The HZAU-HVSEN Research Institute, Wuhan, China

**Keywords:** porcine reproductive and respiratory syndrome (PRRS), tylvalosin, *Poria cocos* polysaccharides (PCP), pig, antiviral, NF-κB, cytokines

## Abstract

Porcine reproductive and respiratory syndrome (PRRS) is one of the most economically important infectious diseases of pigs worldwide. Vaccination and various management measures have been implemented to control PRRS. However, due to high genetic diversity and insufficient understanding of the pathogenesis and immunological mechanisms, PRRS is still a challenge to the pig industry. Therefore, it is important to develop novel strategies to combat PRRS virus (PRRSV) infection. In this study, our data show that tylvalosin, a third-generation animal-specific macrolide, could inhibit PRRSV replication in MARC-145 cells, and suppress the PRRSV-induced NF-κB activation and cytokines expression. The pig infection experiment further demonstrated that tylvalosin could significantly reduce the virus loads in serum and tissues, and alleviate lung lesions of pigs infected with highly pathogenic PRRSV strains. The fever and loss of daily gain (LoDG) of the pigs were decreased as well. Considering the feature of immune suppression of PRRSV, a combination of tylvalosin with the immunopotentiator *Poria cocos* polysaccharides (PCP) was developed. Pig experiment showed this combination had a better therapeutic efficacy against PRRSV infection than tylvalosin and PCP alone in attenuating lung lesions, alleviating fever, and suppressing cytokines production. This study suggests that tylvalosin has significant antiviral and anti-inflammatory effects against PRRSV infection, and the combination of tylvalosin and PCP provides a promising strategy for PRRS treatment.

## Introduction

Porcine reproductive and respiratory syndrome (PRRS) is characterized by reproductive failure in sows and respiratory disorders and reduced growth performance in piglets. This infectious disease was first recognized in the late 1980s in the United States (1) and in the early 1990s in Europe (2). Since then, it became endemic at the global level and causes significant economic losses (3, 4). It has been estimated that PRRS causes $664 million of economic loss a year, or $1.8 million a day in the United States (5), and €126 economic loss per sow per outbreak in the Netherlands (6). China is the world’s largest country of pig farming, and PRRS causes at least $3,500 million, or 24.5 billion RMB of economic loss every year. Controlling PRRS is of great significance for the pig industry worldwide.

One important feature of porcine reproductive and respiratory syndrome virus (PRRSV) infection is the damage to host immune systems, leading to immune suppression. The virus can replicate in bone marrow cells, causing bone marrow hypoplasia manifested by a decrease in total white blood cells (7, 8). PRRSV can also target the thymus and disturb its functions (9). As a result, delayed or inhibited induction of type I and type II interferons (IFNs) was commonly observed after PRRSV infection (10, 11). Due to the suppression of host immune functions caused by PRRSV infection, secondary infections are common, leading to more severe tissue lesions (12, 13). PRRSV can also cause severe pneumonia in neonatal pigs (14). This is associated with the production of inflammatory cytokines, such as IL-1, IL-6, and IFN-γ (15, 16).

PRRSV has a high genetic diversity. It has two genotypes (type 1 and type 2). Type 1 PRRSV, first documented in the early 1990s in Europe (2), is further divided into three subtypes. Type 2 PRRSV, first reported in the late 1980s in North America (1), has evolved into 9 lineages (17–19). The virulence and antigenicity also vary among different types and lineages of PRRSV. The outbreak of PRRS was first reported in 1995 in China, and a highly pathogenic PRRSV (HP-PRRSV) variant, represented by the JXA1 strain, was reported in 2006 and caused devastating damage to the pig industry, which affected over 2 million pigs with about 400,000 deaths (20). A moderately virulent strain NADC30 was isolated in 2008 in the United States (21). Since 2013, diverse NADC30-like strains resulting from recombination between NADC30 and other strains became prevalent in China, which can cause a case-fatality rate of 30 to 50% (22–24).

Currently, vaccination is a widely used approach to prevent PRRSV infection. Both killed vaccines and modified live virus (MLV) vaccines have been developed and some of them have been commercialized. Although these vaccines can alleviate clinical symptoms as well as provide protection against lethal challenges, they also have disadvantages (25). Killed vaccines are safe but can only provide limited protection against homologous and heterologous protection (26). MLV vaccines have better efficacy of protection against homologous viruses and also have partial heterologous protection, therefore are widely used (25, 27). But they have safety concerns and limited protection against emerging field strains (28–30). Hence, alternative strategies to combat PRRSV are needed. It has been reported that host cell surface proteins, such as CD163, mediate PRRSV infection, and CD163 knockout pigs show resistance to PRRSV infection (31, 32), indicating blocking PRRSV entry can be a promising strategy to prevent the infection. MicroRNAs are deemed as another candidate of anti-PRRSV infection strategies. miRNA targeting the PRRSV genome as well as host factors showed antiviral efficacy (33–35). Nanobodies targeting viral proteins, herbal extracts, and chemical compounds have also been shown to have anti-PRRSV effects (36).

Developing novel antiviral drugs is of high cost and time-consuming. Drug-repurposing which develops existing drugs for new indications becomes a promising strategy to screen for antiviral compounds (37). Since the safety and efficacy data of existing drugs are already known, drug-repurposing largely cuts down the developing cost and accelerates the research and development of new drugs (38). Macrolide antibiotics are a group of antibiotics containing a macrocyclic lactone ring, which are widely used in animal and human healthcare. Several members of macrolides are approved antibiotics for use in farm animals, including tylosin, tilmicosin, tylvalosin, tildipirosin, and tulathromycin. They have general activity against mycoplasma and Gram-positive bacteria, and can also inhibit some Gram-negative bacteria. Tylvalosin is a third-generation animal-specific macrolide antibiotic with broad-spectrum antibacterial activities. It has lower toxicity and can rapidly concentrate in various host cells, especially in porcine alveolar macrophages, the major target cells of PRRSV (39). Interestingly, macrolides have been reported to have immune-modulatory effects and several studies have demonstrated that macrolides show efficacy against the infection of viruses, such as PRRSV, herpes simplex virus, and dengue virus (40–43). For example, Zhao et al. showed that tylvalosin exhibits anti-inflammatory effects and alleviates the lung injury caused by PRRSV infection in mice (43).

Traditional Chinese medicines (TCM) provide an alternative option in the prevention and treatment of infectious diseases (44). Studies have proven the efficacy of several TCM in the treatment and prevention of viral infection (45, 46). Polysaccharides are one of the active components of several TCMs. *Poria cocos* polysaccharides (PCP) which are extracted from the TCM *Poria cocos* have been widely used in human medicine. Studies have shown that PCP has a significant immune-modulatory effect (47–49).

Considering the key features of PRRSV infection, including immune suppression and inflammation-induced lung lesion, a novel anti-PRRS strategy was developed by using a combination of tylvalosin and PCP. Our study demonstrated that tylvalosin can inhibit PRRSV replication *in vitro* and *in vivo*, and alleviate PRRSV-induced inflammatory responses and lung lesions, while the PCP can also reduce inflammation and lung lesions of PRRSV-infected pigs. The combination of tylvalosin and PCP provides better therapeutic efficacy than using tylvalosin or PCP alone.

## Materials and methods

### Cells, viruses, and chemicals

MARC-145 cells (ATCC CRL-12231) are a PRRSV permissive cell line derived from African green monkey kidney cells, which were grown in Dulbecco’s modified Eagle’s medium (DMEM) supplemented with a 10% fetal bovine serum (FBS, Gibco) at 37°C under a humidified atmosphere of 5% CO_2_ (50). The JXA1-like PRRSV strain CH-YY (GenBank Accession No. MK450365) and NADC30-like PRRSV strain CH-WH-2019-1 (GenBank Accession No. MK450333) were used in this study. Tylvalosin tartrate (TVN; ≥ 85%) was purchased from the three major producers A, B, and C in the world. *Poria cocos* polysaccharides (PCP; ≥ 90%) powder was provided by Wuhan HVSEN Biotechnology Co., Ltd. (Wuhan, China). MTT Cell Proliferation and Cytotoxicity Assay Kit were purchased from Beijing Solarbio Science & Technology (Beijing, China). Caspase 3 Activity Assay Kit was purchased from Beyotime Institute of Biotechnology (Shanghai, China). Transcriptor First Strand cDNA Synthesis Kit and SYBR Green Master (2×) with ROX were purchased from Roche (Mannheim, Germany). Porcine IL-6, IL-8, and TNF-α enzyme-linked immunosorbent assay (ELISA) kits were purchased from NeoBioscience Technology (Shenzhen, China). The primers used in this study are listed in Table 1.

**Table 1:** List of primers for qRT-PCR.

Primer	Sequence (5′-3′)
PRRSV-N-F	TCAGCTGTGCCAAATGCTGG
PRRSV-N-R	AAATGGGGCTTCTCCGGGTTTT
*Sus scrofa*-GAPDH-F	CCCCAACGTGTCGGTTGT
*Sus scrofa*-GAPDH-R	CCTGCTTCACCACCTTCTTGA
*Sus scrofa*-IL-6-F	TGCTTCTGGTGATGGCTACTG
*Sus scrofa*-IL-6-R	TCCGGAGAGGTGAAGAGCAT
*Sus scrofa*-TNFα-F	CCTTCCACCAACGTTTTCCT
*Sus scrofa*-TNFα-R	TCTGGCAAGGGCTCTTGATG
*Sus scrofa*-IL-1β-F	CCAGGACAAAGACCACAAA
*Sus scrofa*-IL-1β-R	GCAGAACACCACTTCTCTCT
*Sus scrofa*-RANTES-F	CCCACCTCCAGGAATATTTC
*Sus scrofa*-RANTES -R	TTCTCTGGGTTGGCACACA
Macaca-GAPDH-F	TCATGACCACAGTCCACGCC
Macaca-GAPDH-R	GGATGACCTTGCCCACAGCC
Macaca-IL-6-F	GCTGCAGGCACAGAACCA
Macaca-IL-6-R	AAAGCTGCGCAGGATGAGA
Macaca-IL-8-F	CTGGCGGTGGCTCTCTTG
Macaca-IL-8-R	CCTTGGCAAAACTGCACCTT
Macaca-TNFα-F	TCCTCAGCCTCTTCTCCTTCCT
Macaca-TNFα-R	ACTCCAAAGTGCAGCAGACAGA

### Cytotoxicity assay

To investigate the effects of TVN on cell viability, MARC-145 cells (180 μL/well) were cultured in 96-well plates at seeding densities of 2 × 10^5^ cells/ml followed by incubation with 100 μL of TVN at different concentrations (0, 2, 10, 25, and 50 μg/mL) for 3 h. Medium supernatants were removed, and cell viability and apoptosis were detected with the MTT and caspase-3 assays, respectively, according to the manufacturer’s instructions.

### *In vitro* PRRSV inhibition assay

MARC-145 cells were infected with the PRRSV CH-YY strain at a multiplicity of infection (MOI) of 0.01. After 60 min incubation, the medium was replaced with fresh DMEM supplemented with different concentrations (0, 2, 10, 25, and 50 μg/mL) of TVN. At 24 h post-infection, the culture was collected for determination of infectious virus yields by 50% tissue culture infectious dose (TCID_50_) assay. All experiments were done in triplicate.

### Animal experiments

All animal experiments were approved by the Laboratory Animal Monitoring Committee of Huazhong Agricultural University and performed according to the recommendations in the Guide for the Care and Use of Laboratory Animals of Hubei Province, China (HZAUSW-2018-005). The pigs were obtained from a commercial farm, housed in isolated rooms at the animal facilities of the university, and were under the supervision of a veterinarian. Throughout the study, all pigs received feed and water *ad libitum*. The piglets were numbered by ear-tagging and acclimatized for 1 week before experiments.

In the experiment to determine the *in vivo* efficacy of TVN against PRRSV infection, a total of twenty-five 25-day-old weaning piglets (average body weight 6.83 kg) were prescreened and found to be negative for PRRSV by RT-PCR and ELISA. They were randomly divided into the following five groups (5 piglets in each group). Group I was fed with normal diets for 7 days and then intramuscularly and intranasally infected with 3 × 10^5^ TCID_50_ of the JXA1-like PRRSV strain CH-YY (PRRSV CH-YY). Group II was fed with normal diets supplemented with 20% TVN premix (5 mg/kg·bw) for 7 days and then infected with the CH-YY strain in the same way (PRRSV+TVN). Group III was fed with normal feed for 7 days and then infected with 3 × 10^5^ TCID_50_ of NADC30-like strain CH-WH-2019-1 (PRRSV CH-WH-2019-1). Group IV was fed with normal feed supplemented with 20% TVN premix (5 mg/kg·bw) for 7 days and then infected with the CH-WH-2019-1 strain in the same way (PRRSV CH-WH-2019-1 + TVN). Group V was used as a control without any infection or treatment (Mock). After PRRSV infection, the pigs in groups II and IV were fed with normal diets supplemented with 20% TVN premix (5 mg/kg·bw) for 9 days, and the pigs in groups I, III, and V were continuously fed with normal diets. The rectal temperature was measured at 0, 3, 6, and 9 days post-challenge (dpc) for all the five groups of pigs, and the body weight was determined at 4 dpc and 9 dpc, respectively. At 9 dpc, all the pigs were sacrificed and subject to necropsy. The PRRSV loads in the serum and tissue samples were determined by using qRT-PCR as described below.

In the experiment to evaluate the efficacy of the combination of TVN and PCP against PRRSV infection, a total of 35 PRRSV-negative 25-day-old weaning piglets were randomly divided into seven groups. Group I was fed with normal feed for 7 days and then intramuscularly and intranasally infected with 3 × 10^5^ TCID_50_ of CH-YY strain (PRRSV). Group II was fed with normal feed supplemented with 20% TVN premix (5 mg/kg·bw) for 7 days and then infected with the CH-YY strain in the same way (PRRSV+TVN). Group III and group IV were fed with normal feed supplemented with PCP powder (7 and 21 mg/kg·bw, respectively) for 7 days and then infected with CH-YY strain in the same way (PRRSV+PCP7 and PRRSV+PCP21). Group V and group VI were fed with normal feed supplemented with 20% TVN premix (5 mg/kg·bw) as well as the PCP powder (7 and 21 mg/kg·bw, respectively) for 7 days and then infected with CH-YY strain in the same way (PRRSV+TVN + PCP7 and PRRSV+TVN + PCP21). Group VII was used as a control without treatment and infection (Mock). After PRRSV infection, the pigs were fed with the same feed and supplementation in each group as described above for 9 days. The rectal temperature was measured at 0, 3, 6, and 9 dpc, and the body weight was determined at 4 dpc and 9 dpc, respectively. At 9 dpc, all the pigs were euthanized with an intracardiac injection of sodium pentobarbital. The lung tissues were fixed with formaldehyde and subjected to histopathological examinations with regular H & E staining. The Madec-Kobisch lung lesion score was estimated as described previously (51).

### RNA extraction and qRT-PCR analysis

The cell cultures collected from the *in vitro* PRRSV inhibition assay were mixed with Trizol reagent for RNA extraction. At pig necropsy, tissues were homogenized in Trizol reagent for RNA isolation. Then, the total RNA (1 μg) was reverse transcribed to cDNA, and quantitative RT-PCR (qRT-PCR) analysis was performed by using SYBR Mixture with ROX (Applied Biosystems) on the ViiA™ 7 Real-Time PCR System (Applied Biosystems). The amplification conditions were as follows: 60 s at 94°C for pre-denaturation, followed by 40 cycles at 94°C 15 s and 56°C30 s, and fluorescence signals were collected at 56°C. The standard was PCAGs-ORF7 plasmid with 1 × 10^10^ – 1 × 10^1^ copy/ml. All samples were tested in triplicate, and the GAPDH expression level was used as normalization. Relative fluorescence quantitative PCR primers were listed in Table 1. The viremia level was measured by qRT-PCR as described previously (52).

### Statistical analysis

Quantitative data are given as mean values ± SD or as indicated. For analysis of differences between the groups, ordinary one-way analysis of variance (ANOVA) followed by the appropriate *post hoc* test for individual comparisons between the groups was performed. * indicats that the *p* value <0.05 and ** indicates that the *p* value <0.01.

## Results

### *In vitro* inhibition of PRRSV replication by tylvalosin

**Figure 1:**
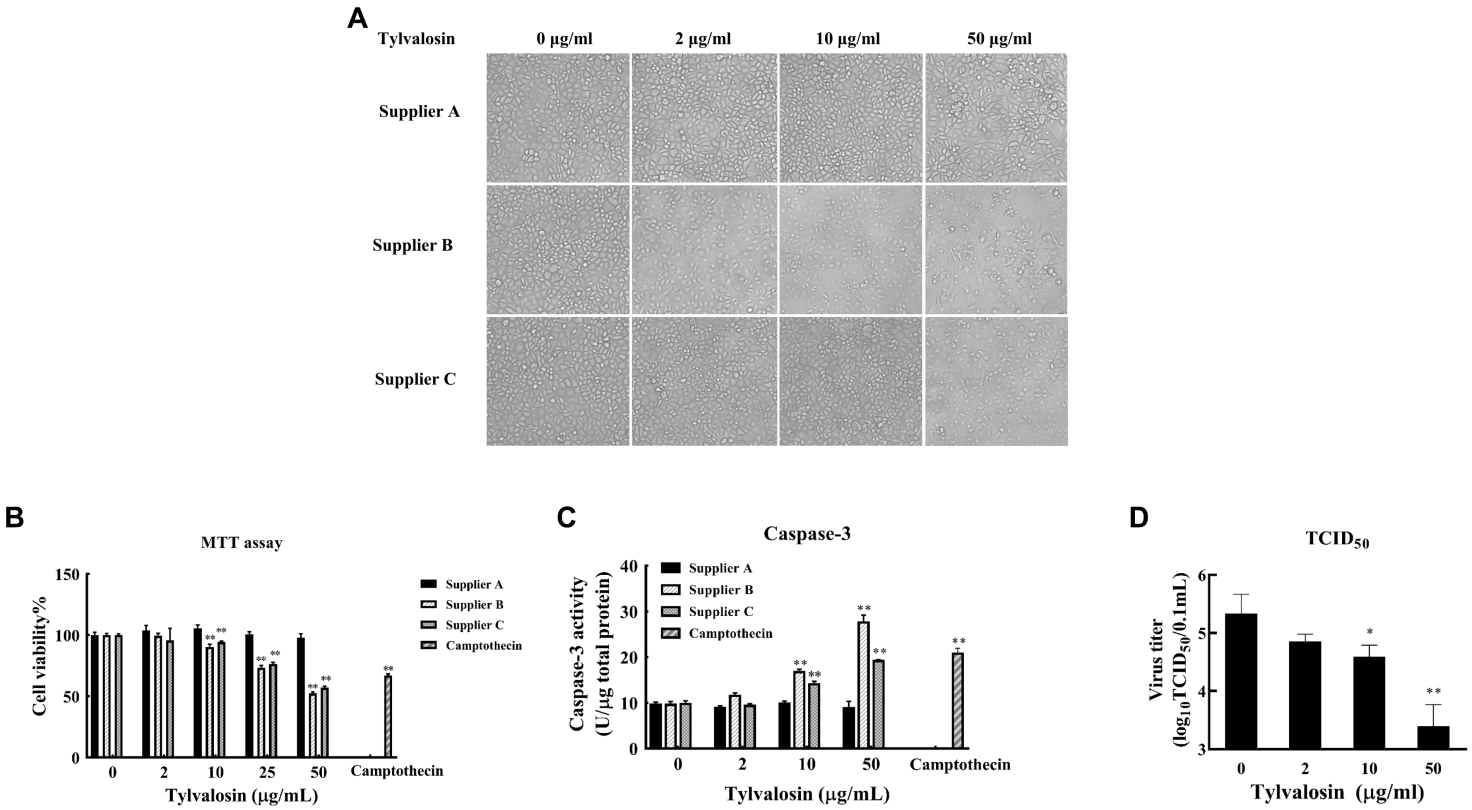
Cytotoxicity and effect on PRRSV replication of tylvalosin. **(A)** Influence of tylvalosin on growth and morphology of MARC-145 cells. Tylvalosin from suppliers A, B, or C was incubated with MARC-145 cells at each indicated concentration. The cells were cultured in normal conditions and imaged after culture for 24 h. **(B)** Cytotoxicity by MTT assay. Tylvalosin from suppliers A, B, or C was incubated with MARC-145 cells at each indicated concentration for 3 h. Camptothecin was used as a positive control. The cytotoxicity was determined using an MTT kit according to the manufacturer’s instructions. The assay was performed in triplicate and the data was shown as the mean ± standard deviation (SD). The statistical significance was calculated using the ANOVA by comparing the data obtained at each indicated time point with that obtained at time point 0 h. **(C)** Caspase-3 activity assay. Tylvalosin from suppliers A, B, or C was incubated with MARC-145 cells at each indicated concentration for 3 h. Caspase-3 activity was measured by using Caspase 3 Activity Assay Kit. The assay was performed in triplicate and the data was shown as the mean ± SD. The statistical significance was calculated using the ANOVA by comparing the data obtained at each indicated time point with that obtained at time point 0 h. **(D)** TCID_50_ assay. MARC-145 cells were infected with PRRSV strain CH-YY and then treated with tylvalosin at each indicated concentration for 24 h. The cell culture was then subjected to TCID_50_ determination. The assay was performed in triplicate and the data was shown as the mean ± SD. The statistical significance was calculated using the ANOVA by comparing the data obtained at each indicated time point with that obtained at time point 0 h. ** indicates that the *p* value <0.01.

The effect of TVN on PRRSV replication in MARC-145 cells was tested. To determine the optimal dose of TVN to use in the *in vitro* assay, the cytotoxicity of TVN purchased from three different suppliers was measured. TVN was incubated with MARC-145 cells with different concentrations and the cell morphology and viability were then analyzed. It was shown that TVN from supplier A had little influence on cell morphology, whereas incubating TVN from suppliers B and C with MARC-145 cells caused abnormal cell morphology when used at 50 μg/mL (Figure 1A). The MTT assay showed that TVN from different suppliers displayed varied toxicity and the one from supplier A did not induce significant cell death even when used at 50 μg/mL (Figure 1B). The Casepae-3 activity in the presence of different concentrations of TVN was also measured. The data showed that TVN from supplier A did not cause activation of Caspase-3 of MARC-145 cells when used at up to 50 μg/mL, while TVN from suppliers B and C could already activate Caspase-3 at 10 μg/mL (Figure 1C). A HP-PRRSV strain CH-YY was used to infect MARC-145 cells which were then treated with different concentrations of TVN from supplier A to see if TVN had any effect on PRRSV replication *in vitro*. TCID_50_ analysis showed that the TVN can significantly inhibit the replication of PRRSV at 10 μg/mL and 50 μg/mL (Figure 1D).

### Tylvalosin suppresses PRRSV-induced NF-κB activation and cytokines production

**Figure 2:**
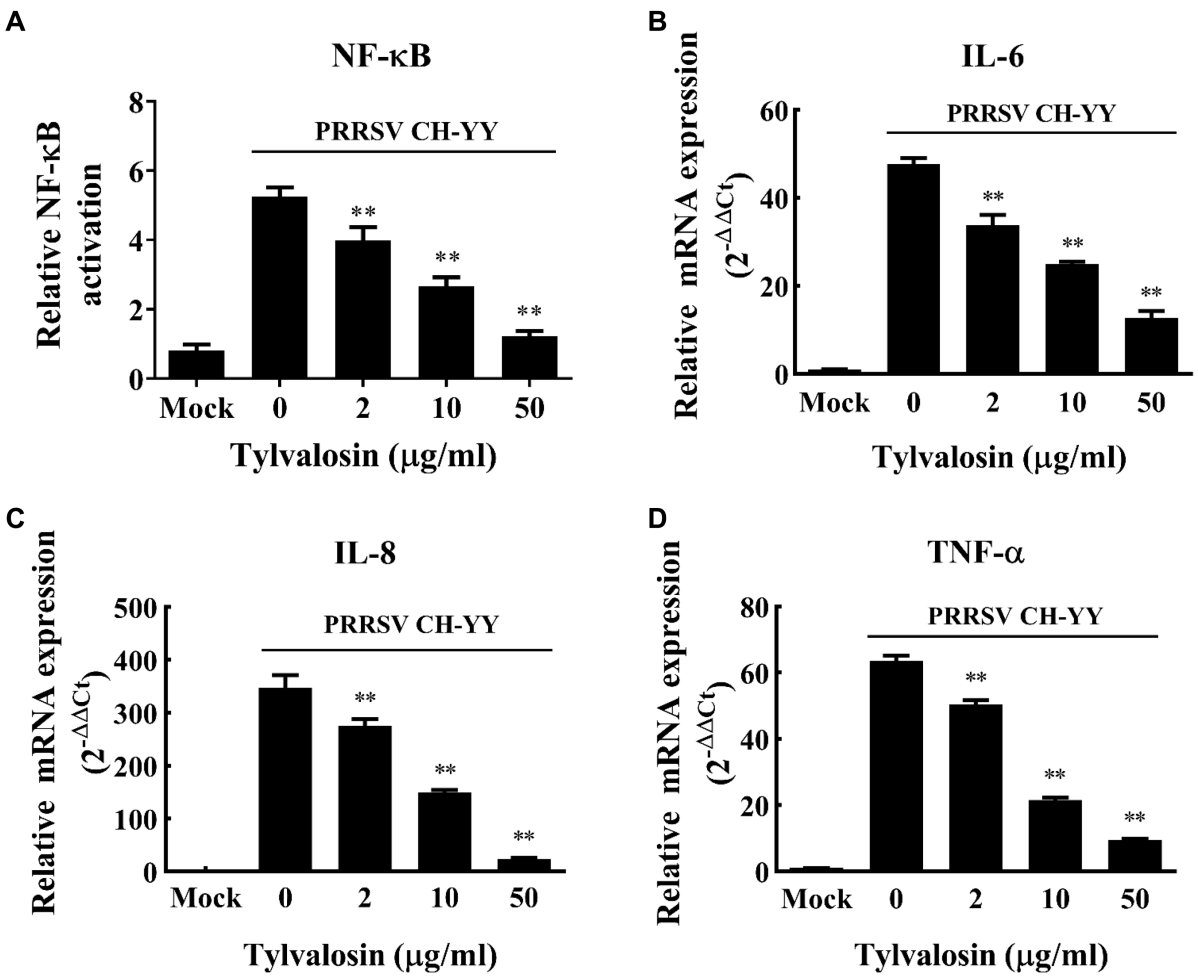
Inhibitory effect of tylvalosin on NF-κB activation and cytokines production induced by PRRSV infection. **(A)** MARC-145 cells seeded in 48-well plate were co-transfected with 50 ng/well of reporter plasmid NF-κB-Luc along with 50 ng/well of pRL-TK. At 24 h post-transfection, the cells were treated with tylvalosin at different concentrations (0, 2, 10, 25, 50 μg/mL) and infected with PRRSV strain CH-YY. The NF-κB activity was determined using a dual-luciferase reporter assay system. **(B–D)** MARC-145 cells were infected by PRRSV strain CH-YY and then treated with TVN. The expression of IL-6 **(B)**, IL-8 **(C)**, and TNF-α **(D)** was determined by qRT-PCR. Mock indicates the samples without PRRSV infection or tylvalosin treatment. The assays were performed in triplicate and the data was shown as the mean ± SD. The statistical significance was calculated using the ANOVA by comparing the data obtained by treatment with each indicated concentration of tylvalosin with that obtained without tylvalosin treatment. ** indicates that the *p* value <0.01.

The inflammatory responses of MARC-145 cells induced by HP-PRRSV infection in the presence and absence of TVN were tested. It was revealed that after infection with the PRRSV CH-YY strain, NF-κB expression was significantly activated (Figure 2A). While when treated with different concentrations of TVN, the PRRSV-activated expression of NF-κB was significantly repressed (Figure 2A). The inhibitory effect showed a dose-dependent manner. Consequently, the production of pro-inflammatory cytokines IL-6, IL-8, and TNF-α was also significantly suppressed (Figures 2B–D). These results suggest that TVN can inhibit PRRSV-induced NF-κB activation and cytokines production.

### Tylvalosin inhibits PRRSV infection in pigs

**Figure 3:**
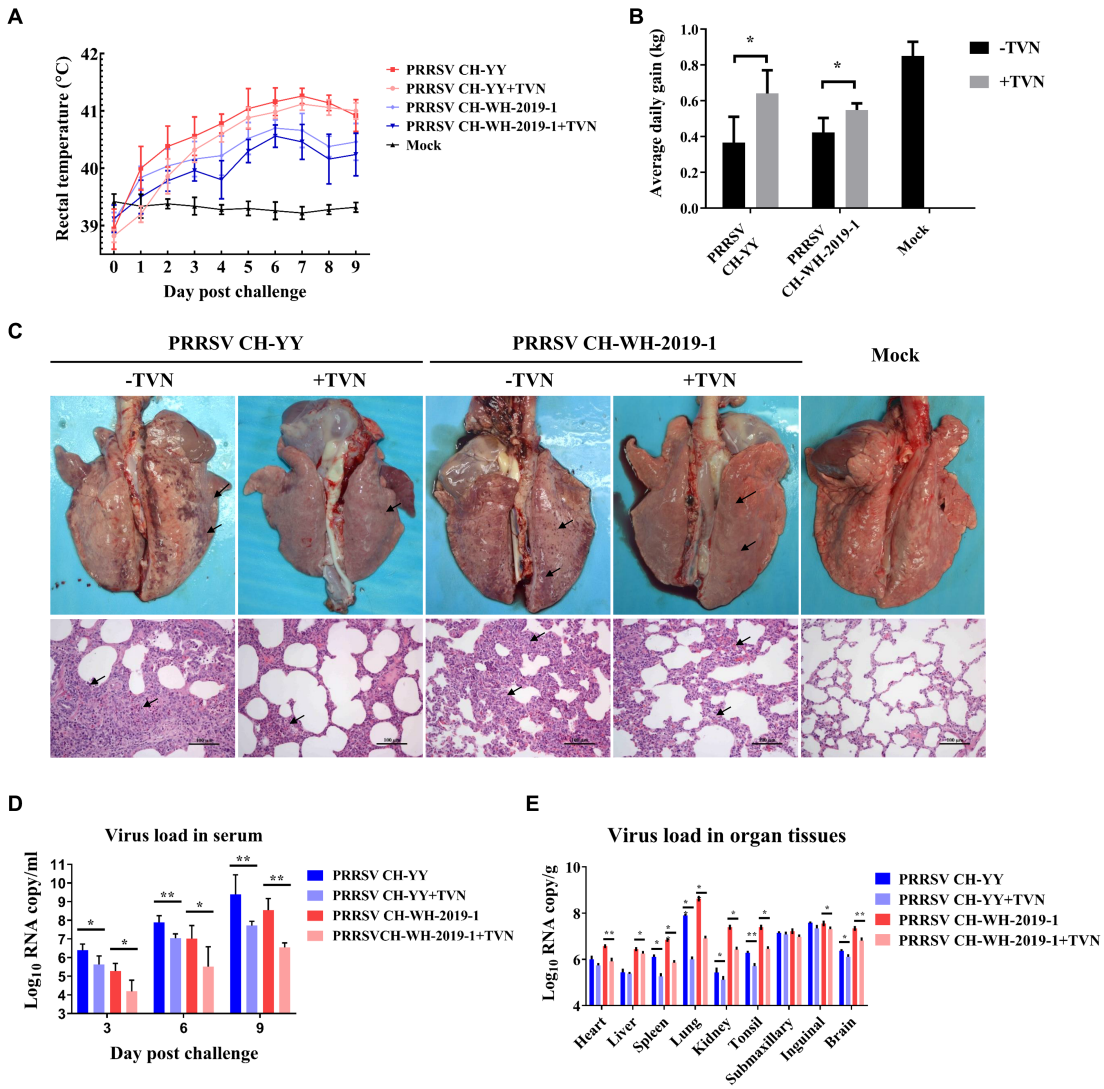
Tylvalosin shows therapeutic efficacy against PRRSV infection in pigs. **(A)** Rectal temperature. The JXA1-like strain CH-YY or NADC30-like strain CH-WH-2019-1 was used to infect pigs treated with or without TVN. The rectal temperature was recorded at each indicated time point. The data was shown as the mean ± SD. **(B)** Average daily gain. The body weight of each pig was monitored and the average daily gain was calculated. **(C)** Gross and histopathological analysis. At the end of the experiment, the pigs were sacrificed and the lungs were collected and imaged (Upper panel). The lung tissues were then fixed and subjected to histopathological analysis (lower panel). The lung lesions are marked with black arrows. The scale bar is 100 μm. **(D)** Virus load in serum. The blood was collected from the pigs at the indicated time point. The serum was isolated and virus load was determined by qRT-PCR. The data were shown as the mean ± SD. The statistical significance was calculated using the ANOVA. **(E)** Virus load in organ tissues. After the piglets were sacrificed, each indicated organ was collected. The total RNA was extracted and the virus load was determined by qRT-PCR. The statistical significance was calculated using the ANOVA. * indicates that the *p* value <0.05. ** indicates that the *p* value <0.01.

The antiviral efficacy of TVN on PRRSV infection in pigs was then evaluated. Two PRRSV strains, the JXA1-like strain CH-YY and the NADC30-like strain CH-WH-2019-1, were used to infect piglets. It was shown that after infection, the pigs displayed a dramatic increase in rectal temperature (Figure 3A). In contrast, TVN treatment could significantly repress the body temperature increase (Figure 3A) and the average daily weight gain was significantly higher in the TVN-treated groups than those without TVN treatment (Figure 3B). Gross anatomy was performed at the end of the experiment and it was demonstrated that both of the PRRSV strains caused severe lung lesion (Figure 3C, marked with black arrows). While TVN treatment significantly alleviated the lesion (Figure 3C). Virus loads in serum and tissues were quantified by real-time RT-PCR. It was shown that from 3 to 9 dpc the virus load in the sera of the TVN-treated pigs was significantly lower than those of the untreated pigs (Figure 3D). At 9 dpc, the virus load in several organ tissues also showed a significant reduction in the TVN-treated pigs (Figure 3E). These results suggest that TVN has an antiviral effect against PRRSV *in vivo*.

### Combination of TVN and PCP provides better efficacy on PRRSV infection in pigs

**Figure 4:**
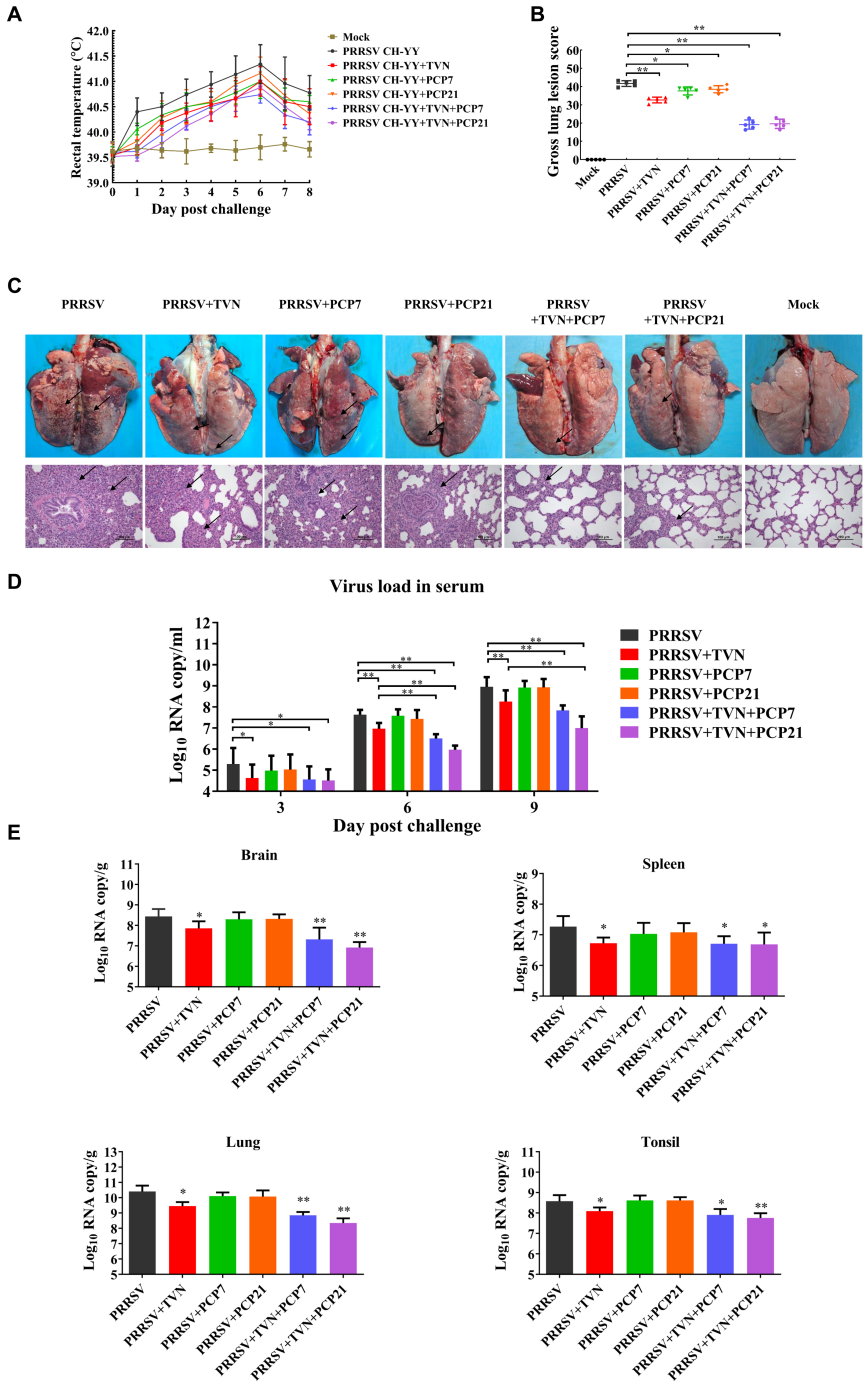
Efficacy of the combination of TVN and PCP on PRRSV infection in pigs. **(A)** Rectal temperature. The rectal temperature was recorded at each indicated time point. The data was shown as the mean ± SD. **(B)** Gross lung lesion score. The pigs were sacrificed at the end of the experiment and the lung lesion was scored according to Halbur et al. (53). **(C)** Gross and histopathological analysis. At the end of the experiment, the pigs were sacrificed and the lung was collected and imaged (Upper panel). The lung tissues were then fixed and subjected to histopathological analysis (lower panel). The lung lesions are marked with black arrows. The scale bar is 100 μm. **(D)** Virus load in serum. The blood was collected from the pigs in each group at the indicated time point. The serum was isolated and virus load was determined by qRT-PCR. The data was shown as the mean ± SD. The statistical significance was calculated using the ANOVA. * indicates that the *p* < 0.05. ** indicates that the *p* < 0.01. **(E)** Virus load in organ tissues. The piglets wereFIGURE 4 (Continued)sacrificed at the end of the experiment and each indicated organ was collected. The total RNA was extracted and the virus load was determined by qRT-PCR. The statistical significance was calculated using the ANOVA. * indicates that the *p* value <0.05. ** indicates that the *p* value <0.01. Mock indicates the group without infection or TVN treatment. PRRSV indicates the group with PRRSV CH-YY infection but without TVN treatment. PRRSV+TVN indicates the group with PRRSV CH-YY infection as well as TVN treatment. PRRSV+PCP7 indicates the group with PRRSV CH-YY infection as well as PCP treatment at 7 mg/kg·bw. PRRSV+PCP21 indicates the group with PRRSV CH-YY infection as well as PCP treatment at 21 mg/kg·bw. PRRSV+TVN + PCP7 indicates the group with PRRSV CH-YY infection as well as TVN treatment and PCP treatment at 7 mg/kg·bw. PRRSV+TVN + PCP21 indicates the group with PRRSV CH-YY infection as well as TVN treatment and PCP treatment at 21 mg/kg·bw.

PCP has been shown to have strong immunomodulatory activity. It was next explored whether TVN in combination with PCP was able to further enhance the therapeutic efficacy against PRRSV in pigs. After PRRSV CH-YY infection, the group without any treatment (PRRSV) showed the highest level of body temperature increase, while the PRRSV+TVN + PCP7 and PRRSV+TVN + PCP21 groups showed the lowest levels (Figure 4A). At 9 dpc, all the pigs were sacrificed and necropsy data indicated that the control group (PRRSV) showed the most severe lung lesions (Figure 4C, marked with black arrows). Administration with TVN alone (PRRSV+TVN), PCP alone (PRRSV+PCP7 and PRRSV+PCP21), and a combination of TVN and PCP (PRRSV+TVN + PCP7 and PRRSV+TVN + PCP21) all significant alleviated the lung lesions (Figures 4B,C). By scoring the lesion, it was shown that the combination of TVN with PCP had better efficacy than using TVN or PCP alone (Figure 4B). Further histopathological examinations with the lung tissues showed that infection with PRRSV caused marked thickening of interalveolar septa, septal infiltration of mononuclear cells, and hypertrophy and hyperplasia of alveolar epithelial cells, and the pigs in the PRRSV+TVN + PCP7 and PRRSV+TVN + PCP21 groups showed the mildest lesion in the lungs, which were better than that of the pigs treated with TVN (PRRSV+TVN) or PCP alone (PRRSV+PCP7 and PRRSV+PCP21) (Figure 4C lower panel). Virus loads were then determined in the serum as well as in different organ tissues. It was shown in Figure 4D that following PRRSV infection, PRRSV was detected in the serum of all the infected pigs at 3, 6, and 9 dpc. Notably, TVN treatment significantly decreased the virus load in serum, whereas PCP treatment alone had no effects on decreasing the virus load (Figure 4C). Moreover, at 6 dpc and 9 dpc, the PRRSV+TVN + PCP21 group showed a significantly lower virus load in the serum than that of the PRRSV+TVN group (Figure 4C). In organs including brain, lung, spleen, and tonsil, the virus load was significantly lower in the TVN-treated groups than the control. Also, the groups treated with the combination of TVN and PCP showed a lower virus load in the organ tissues than the groups treated with TVN or PCP alone (Figure 4D). These data indicate that TVN in combination with *Poria cocos* polysaccharides provides better efficacy against PRRSV infection in pigs than using TVN alone.

### Combination of TVN and PCP alleviates inflammatory responses in the pig lung induced by PRRSV infection

**Figure 5:**
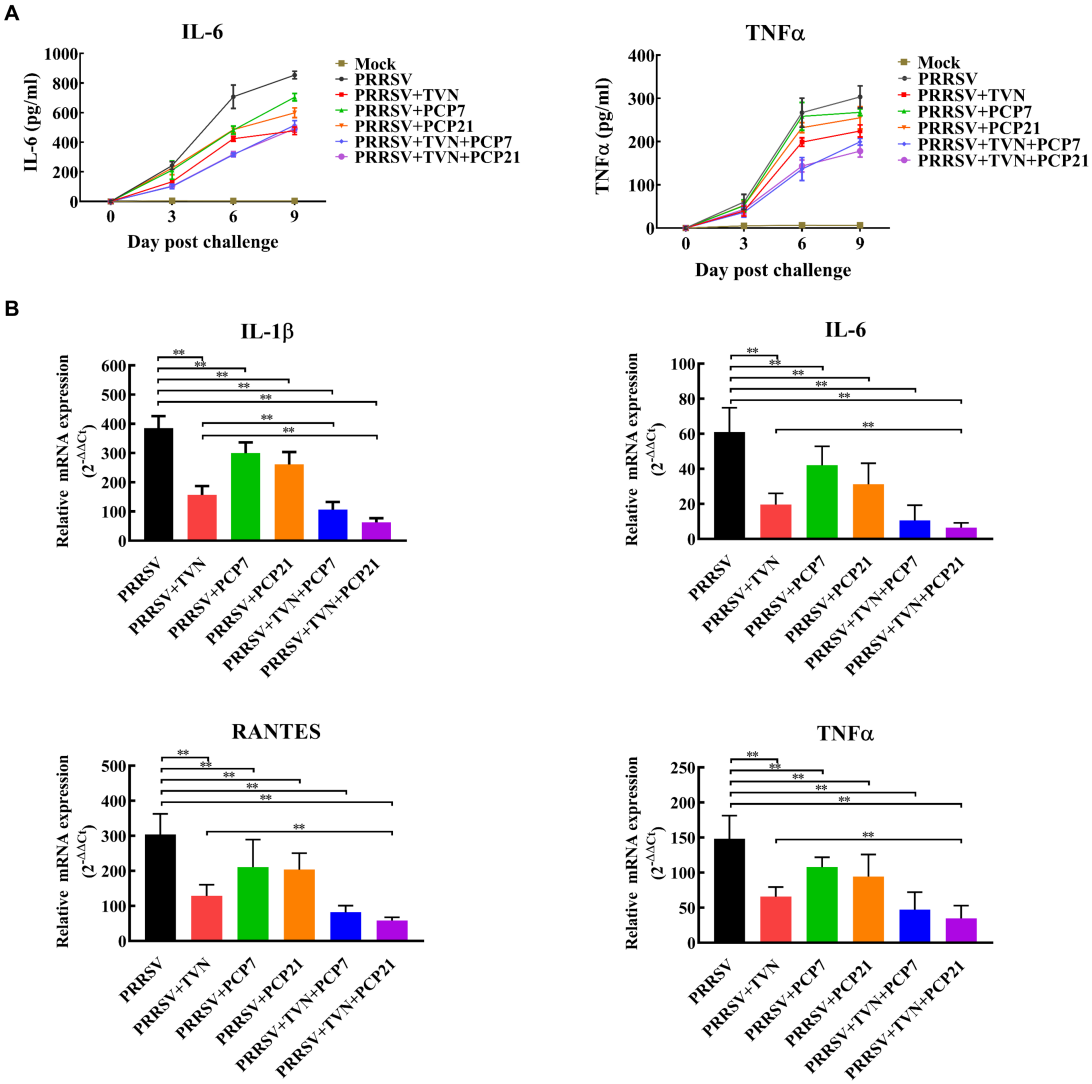
Cytokines quantification. The pigs in each group were sacrificed at the end of the experiment. The lungs were collected. The cytokines in the serum were determined by commercial pig IL-6 and TNF-α ELISA kits **(A)** and those in the lung tissues were determined by qRT-PCR **(B)**. The statistical significance was calculated using the ANOVA. * indicates that the *p* value <0.05. ** indicates that the *p* value <0.01. Mock indicates the group without infection or TVN treatment. PRRSV indicates the group with PRRSV CH-YY infection but without TVN treatment. PRRSV+TVN indicates the group with PRRSV CH-YY infection as well as TVN treatment. PRRSV+PCP7 indicates the group with PRRSV CH-YY infection as well as PCP treatment at 7 mg/kg·bw. PRRSV+PCP21 indicates the group with PRRSV CH-YY infection as well as PCP treatment at 21 mg/kg·bw. PRRSV+TVN + PCP7 indicates the group with PRRSV CH-YY infection as well as TVN treatment and PCP treatment at 7 mg/kg·bw. PRRSV+TVN + PCP21 indicates the group with PRRSV CH-YY infection as well as TVN treatment and PCP treatment at 21 mg/kg·bw.

To further characterize the effects of the combination of TVN and PCP on the inflammatory responses after PRRSV infection, the level of pro-inflammatory cytokines in serum and lung tissue was determined by ELISA and real-time qPCR, respectively. It was shown in Figure 5A that the levels of IL-6 and TNF-α in the serum began to increase following PRRSV infection in all groups. PRRSV+TVN + PCP7 and PRRSV+TVN + PCP21 groups showed the lowest level of IL-6 and TNF-α in the serum and showed statistical significance at 6 dpc than the other groups (Figure 5A). Real-time qPCR further revealed that the combination of TVN and PCP significantly decreased the levels of IL-1β, IL-6, RANTES, and TNF-α in the lung compared to the group treated with PCP or TVN alone (Figure 5B).

## Discussion

PRRSV is one of the leading pathogens that severely impacts pig farming worldwide, resulting in substantial economic losses. Currently, vaccines are widely used to prevent PRRS, and both inactivated and live PRRSV vaccines have been developed and are commercially available (54). Nevertheless, PRRS control is still a big challenge considering the high genetic diversity of the virus and the insufficient understanding of the pathogenesis and immunological mechanisms of PRRSV infection (55–57). In this study, our results demonstrate that a macrolide antibiotic tylvalosin has therapeutic efficacy against PRRSV infection *in vitro* and *in vivo*. Moreover, by combining tylvalosin with the TCM *Poria cocos* polysaccharides, a more effective approach to contain PRRSV infection in pigs is achieved. Our findings have the potential to be advantageous in mitigating economic losses in swine farms that consider treatment in PRRS-infected pigs.

Antiviral drugs are attracting increasing interest. For PRRSV, several compounds or herbal extracts have been shown to have *in vitro* or *in vivo* antiviral efficacy. Atractylodinol and ethoxysanguinarine were reported to have anti-PRRSV activity with an inhibitory concentration of 50% (IC_50_) of 7.9 and 39.4 μM, respectively (58). 3-O-b-chacotriosyl ursolic acid and its ester analogs exhibited potent anti-PRRSV activity *in vitro* with minimal cytotoxicity (59). Nevertheless, there is still a long time before these compounds are further developed into approved drugs since novel drug development is of high cost and risk. Under this circumstance, the strategy that exploits existing drugs for new medical indications, i.e., drug repurposing, becomes a promising option for developing antiviral therapies (60, 61).

Macrolides are a group of antibiotics widely used in human and veterinary medicine to treat bacterial infections. Interestingly, studies have shown that macrolides display other pharmaceutical properties than antibiotic activity (62, 63). Accumulating evidence has suggested that macrolides have immune-modulation activities. For example, azithromycin has been shown to have anti-inflammatory effects by interfering with the expression of inflammatory factors and enhancing macrophage functions (64). Erythromycin can promote the differentiation of monocyte to macrophages (65, 66). Several studies have also reported the antiviral activity of macrolides (67, 68). Tylvalosin is a third-generation macrolide antibiotic. It has lower toxicity and enters host cells rapidly (39). In this study, by performing cell and pig infection experiments, our data show that tylvalosin has *in vitro* and *in vivo* efficacy against PRRSV infection. Consistently, a recent study also revealed the antiviral effect of tylvalosin against PRRSV infection and provided some insights into the underlying mechanism by transcriptomics analysis (69). These studies broaden the application of tylvalosin and provide an alternative choice to treat PPRSV infection.

When evaluating the *in vitro* effect of tylvalosin on PRRSV replication, the cytotoxicity of tylvalosin from three different suppliers was first tested. It was noticed that tylvalosin from suppliers B and C showed toxicity even at 10 mg/mL, while that from supplier A showed little toxicity within 50 mg/mL (Figure 1). This indicates that the clinical efficacy of tylvalosin may vary among different suppliers.

In the *in vitro* and *in vivo* assays to determine the efficacy of tylvalosin against PRRSV infection, it was found that tylvalosin treatment can efficiently decrease the virus loads in the MARC-145 cells and the pig serum (Figures 1, 3). Previous studies have also reported the antiviral activity of macrolide antibiotics. Tilmicosin exhibited strong antiviral effects which might be associated with the alteration of endosomal pH and the disassembly of virions (40). Spiramycin and azithromycin possess antiviral activities against enterovirus and coxsackievirus likely through interfering with viral RNA replication (67, 68).

Our results showed that PRRSV infection induced the activation of NF-κB, which is consistent with previous studies (70, 71). NF-κB is a key transcription factor involved in a variety of inflammatory responses and the expression of antiviral cytokines (72). Upon treatment with tylvalosin, the PRRSV-induced activation of NF-κB was significantly attenuated (Figure 2). This may explain the anti-PRRSV effect since previous studies reported that inhibiting the NF-κB pathway by carbon monoxide or miRNA could reduce PRRSV replication (73, 74). Moreover, it was found that the production of inflammatory cytokines, such as IL-6, IL-8, and TNF-α, was also elevated after PRRSV infection (Figures 2, 5). These cytokines have been believed to contribute to the pathogenesis of PRRSV infection (75, 76). When treated with tylvalosin, the PRRSV-induced production of the cytokines was significantly suppressed (Figures 2, 5). Previous studies have also reported that tylvalosin can induce apoptosis of porcine neutrophils and macrophages and suppresses inflammation (77) and can reduce lipopolysaccharide (LPS)- and PRRSV- induced lung injuries (78).

In this study, our data show that tylvalosin combined with PCP has a better efficacy against PRRSV infection in the pig infection assay. PCP is extracted from *Poria cocos* and has been reported to have potent immune-enhancing activities. It can increase antigen-specific antibody levels following immunization with the influenza vaccine as well as promote the proliferation of splenocytes and IL-12p70 and TNF-α production in mice, indicating an enhancing effect on both humoral and cellular immunity (47). Zhang et al. also reported that PCP can enhance rabies vaccine-induced immune responses (79). Moreover, PCP is reported to have an anti-inflammatory effect. It can inhibit the production of several inflammatory cytokines induced by LPS stimulation through the inactivation of the NF-κB pathway in the RAW 264.7 cells (80). In our results, PCP alone did not reduce the virus load in serum or organ tissues of the PRRSV-infected pigs (Figure 4F), whereas it significantly suppressed the production of the cytokines (Figure 5) and alleviated the lung lesion (Figures 4C–E). Notably, the group treated with tylvalosin in combination with PCP (TVN + PCP21) exhibited the lowest lung lesion, virus load, and pro-inflammatory cytokines expression levels in the pig infection assay (Figures 4, 5). As PRRSV infection is featured by immune suppression and lung lesions through hyperinflammation, the combination of tylvalosin and PCP can provide better efficacy.

## Data availability statement

The original contributions presented in the study are included in the article/supplementary material, further inquiries can be directed to the corresponding author.

## Ethics statement

The animal study was approved by the Laboratory Animal Monitoring Committee of Huazhong Agricultural University. The study was conducted in accordance with the local legislation and institutional requirements.

## Author contributions

RZ, JL, and WZ conceived and supervised the research. HS, WL, SW, JD, and CC performed research. HS, SL, and QH analyzed data. HS, QH, and RZ wrote the manuscript. All authors contributed to the article and approved the submitted version.

## Funding

This research was supported by the Hubei Provincial Science and Technology Innovation Project (No. 2019AEE005).

## Conflict of interest

The authors declare that the research was conducted in the absence of any commercial or financial relationships that could be construed as a potential conflict of interest.

## Publisher’s note

All claims expressed in this article are solely those of the authors and do not necessarily represent those of their affiliated organizations, or those of the publisher, the editors and the reviewers. Any product that may be evaluated in this article, or claim that may be made by its manufacturer, is not guaranteed or endorsed by the publisher.
